# Convolutional Neural Network-Based Discriminator for Outlier Detection

**DOI:** 10.1155/2021/8811147

**Published:** 2021-03-02

**Authors:** Fahad Alharbi, Khalil El Hindi, Saad Al Ahmadi, Hussien Alsalamn

**Affiliations:** Department of Computer Science, College of Computer and Information Sciences, King Saud University, Riyadh 11543, Saudi Arabia

## Abstract

Noise in training data increases the tendency of many machine learning methods to overfit the training data, which undermines the performance. Outliers occur in big data as a result of various factors, including human errors. In this work, we present a novel discriminator model for the identification of outliers in the training data. We propose a systematic approach for creating training datasets to train the discriminator based on a small number of genuine instances (trusted data). The noise discriminator is a convolutional neural network (CNN). We evaluate the discriminator's performance using several benchmark datasets and with different noise ratios. We inserted random noise in each dataset and trained discriminators to clean them. Different discriminators were trained using different numbers of genuine instances with and without data augmentation. We compare the performance of the proposed noise-discriminator method with seven other methods proposed in the literature using several benchmark datasets. Our empirical results indicate that the proposed method is very competitive to the other methods. It actually outperforms them for pair noise.

## 1. Introduction

While the effectiveness of supervised machine learning algorithms relies on the existence of large and high-quality labeled datasets, it is a time-consuming and challenging matter to create clean datasets that are free from noise (i.e., incorrectly labeled instances) [1, 2]. Outliers (noise and outlier are used interchangeably in this paper to refer to the mislabeled instances) occur in real-world datasets for many reasons that are related to data collection, human errors, and the widespread use of suboptimal automated processes to compile large datasets. The aim of this research is to propose a machine learning method for identifying and eliminating noise from datasets. We propose a method to train a noise discriminator (ND). The ND is trained using automatically generated datasets based on a small number of genuine instances. The NDs that we propose are CNN classifiers.

Deep learning (DL) models, including CNN, have been applied with great success in diverse areas with a performance that often exceeds the capabilities of human beings [3, 4]. DL models are particularly valuable in domains where large amounts of training data are available. However, when a training dataset's size increases, so does the likelihood that it contains outliers, leading ML models to overfit the training data, thereby undermining the performance [5]. Given the negative effects of outliers on DL methods, a range of solutions has been identified to mitigate these effects [6].

This research focuses on developing a generalized CNN-based discriminator for outlier identification. The proposed method trains the discriminator on a specially built dataset, generated from a small number of genuine instances. The discriminator can be used as a preprocessing step to identify and eliminate outliers prior to their use to train classifiers.

Our proposed method was inspired by the generative adversarial network (GAN) model [7] that contains a discriminator model that is trained to separate genuine images from fake images produced by a generator. Similarly, we build a noise discriminator that can identify outliers based on preprepared genuine data (noise free). However, unlike GAN, we do not have a generator model; rather, we systematically generate the training data. The proposed discriminator is trained using a dataset derived from the trusted data. The training dataset contains all the possible mislabeled cases. In other words, the discriminator will be trained using every genuine instance and all of its potential mislabeling.

The remainder of this paper is organized as follows.  Section 2 presents an overview of the related works that deal with outliers. In Section 3, the proposed discriminator is presented.  Section 4 discusses the empirical results used for evaluating the performance of the proposed discriminator. Finally, Section 5 is the conclusion.

## 2. Related Work

Various studies have been conducted to develop methods for mitigating the effect of mislabeled instances. Some of those methods are based on using an improved loss function that is robust against mislabeling, such as [8, 9]. Other methods, such as [10, 11], use instance reduction techniques to reduce the size of the training set and eliminate noise.

Another approach for handling noise is based on using the trusted data. Zeng and Martinez [12] proposed a neural network-based noise filter for a pretraining process to reduce the effect of noise in the nearest neighbor classifiers. Their mechanism was based on using a class probability vector that is updated consistently during training for each instance. The class probability vector is updated using a learning algorithm that is based on the disparity between its value and the neural network prediction. A potential limitation of this approach is that it requires training the neural network using a sufficient number of correct instances that may not always be available.

Sukhbaatar and Fergus [13] developed a framework in the context of neural networks that includes an extra noise layer on top of the network to handle the noisy labels. They proposed bottom-up and top-down noise models. In the bottom-up model, the label probability output is varied according to the noisy labels. In contrast, the top-down model is used for modifying the noisy labels before using them for training. They introduced a technique for estimating the noise distribution using clean and noisy data. This technique involves building two confusion matrices for both clean and noisy data; the difference between these two confusion matrices represents the noise distribution. They also found that reducing the weight of the noisy data is very effective.

The concept of distillation, Hinton et al. [14], involves training one model using the knowledge transferred from another model. This concept inspired Li et al. [15] to develop a framework that can learn from noisy labels. A model is trained using a small clean dataset, and the learned knowledge is transferred for training another model on large, noisy datasets. However, obtaining a sufficient number of clean data remains a challenge for this method. Hendrycks et al. [16] proposed Gold Loss Correction, a loss function for handling noise. The proposed method is based on the availability of trusted data that is used for estimating a matrix *C* of corruption probabilities $C_{ij}=\rho \left(\tilde{𝒴}=j|𝒴=i\right)$; the matrix is *K* × *K*, where *K* represents the number of classes in a dataset. The matrix *C* is then used to train a classifier that is expected to be able to predict correct labels. However, the minimum genuine data required were 5%, which may be too large in datasets such as MNIST [17], CIFAR-10 [18], and SVHN [19].

Various methods for handling noise have also been proposed in the context of CNN training [6, 20]. A probabilistic graphical model was integrated by Xiao et al. [21] into a deep learning framework to schematize the relationships between the input, class labels, and noisy labels in a clothing dataset. They utilized the expectation-maximization algorithm in the training process. JoCoR [22] is a method that utilizes two networks that are trained simultaneously. During every batch of training, each network feeds the other by supposedly clean instances during training, where clean instances are identified by having small-loss values. The two networks are implemented with one regularization term to reduce the diversity between the two networks. Another method called EBF [23, 24] is used as a filtration technique during the training of neural network-based models. It detects and removes noisy instances based on the exponential moving average (EMA) of the loss values for instances. The assumption is that any EMA of an instance that exceeds a certain threshold represents noise. In turn, elimination occurs during the training procedure.

The GAN model [7] contains two submodels, a generator, and a discriminator. The generator model is trained to produce fake images that are assumed to be similar to the original ones, while the discriminator is trained to be able to determine if an image is original or fake. During training, the generator and the discriminator are locked in a contest, each attempting to beat the other one until the generator can produce fake images that the discriminator cannot identify.

In this paper, we propose a discriminator trained on a dataset produced using a few genuine (trusted) data for identifying mislabeling instances.

## 3. Discriminator for Outlier Detection

**Figure 1:**
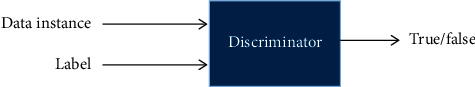
Discriminator input and output.

Machine learning (ML) methods seek to find a good approximation of a target function that maps an input, *x*, into an output, *y* [5]. Thus, ML algorithms learn a function, *f*(*x*), that can predict an output *y* for any given input *x*. This section presents a method for constructing classifiers that can identify outlier instances in a noisy dataset. Specifically, the proposed method involves building a discriminator model for outlier detection that can be used for data cleansing. The discriminator receives an input instance and a possible label, after which it seeks to determine whether the label is correct or not, as shown in Figure 1.

### 3.1. Generating the Training Data

The noise discriminator is trained using a training dataset that is generated based on a small number of trusted (genuine) instances; we call them the seeds. We use the trusted instances to create a dataset that contains instances labeled with either 1 or 0. An instance has label 1 if it is a genuine instance (not noisy) and label 0 if it is a noisy instance. To create noisy instances, we populate the dataset with instances labeled with all classes other than their correct class. Genuine instances are inserted and replicated to ensure that the dataset is balanced.

The discriminator is trained using the generated dataset $\tilde{D}$, where $\tilde{D}$ is derived from a small set of data, *D*_Trusted_. For every genuine instance of the form 〈*x*, *y*〉, in the trusted dataset, *D*_Trusted_, we derive *K* instances and insert them in $\tilde{D}$, where *K* is the number of classes. For every class *c* and a genuine instance, 〈*x*, *y*〉, $\tilde{D}$ is augmented with one instance of the form ≪*x*, *y* > , 1> and *K* − 1 instances of the form ≪*x*, *c* > , 0>, where *c* ≠ *y*. In $\tilde{D}$, if an instance has label 1, this indicates that it is a genuine instance with the correct class; however, if an instance has label 0, this indicates that it is a mislabeled instance (an instance with an incorrect class).

Considering the example of the digit recognition problem [25], for every seed (genuine) instance, $\tilde{D}$ will contain ten instances: one instance of the form ≪*x*, *y* > , 1> representing that *y* is the correct digit of *x* and nine instances of the form ≪*x*, *c* > , 0> representing the fact that *c* is not the correct digit of *x*. These nine instances cover all possible mislabeling of *x*.  Table 1 illustrates the instances that will be added to $\tilde{D}$ for a genuine instance (image) of digit two. This means that, for every seed instance, $\tilde{D}$ will be augmented by one instance of class 1 and nine instances of class 0.

To ensure that the resulting dataset is balanced, each instance of class 1 in $\tilde{D}$ is duplicated *K* − 2 times. This is a form of oversampling to balance the training set.  Algorithm 1 provides a detailed account of the procedure for creating $\tilde{D}$.

The size of $\tilde{D}$ (i.e., number of instances) can be calculated as follows:

(1) $$\begin{array}{c} \text{number of instances in }\tilde{D}=G\times K\times 2\left(K-1\right), \end{array}$$

where *G* represents the number of seed instances per class. In addition, data augmentation methods can be used to generate more training data and avoid overfitting. For instance, operations such as zooming, rotation, shearing, and shifting are commonly used methods in image recognition systems [26]. Therefore, we apply the following data augmentation procedures: firstly, shifting the width and height by 0.2; secondly, rotating by 50 degrees; thirdly, zooming by 0.9 for width and 0.8 for height; and finally, shearing with the transformation intensity of 0.2. The size of $\tilde{D}$ (i.e., number of instances) in case of using data augmentation methods can be obtained as follows:

(2) $$\begin{array}{c} \text{no}.\text{ of instances in }\tilde{D}=G\times K\times 2\left(K-1\right)\times \text{AugFact}\times DA, \end{array}$$

where AugFact is the number of times we perform data augmentation and *DA* denotes the number of augmented images produced from each original image (including the original ones).

### 3.2. A Convolutional Neural Network-Based Discriminator

In this section, we discuss the architecture of the noise discriminator. It is a CNN-based architecture that contains some convolutional and pooling layers. The convolutional layers are an effective model for feature extraction [27]. In particular, CNN comprises multiple convolution layers where the output of each layer is fed as input for the next layer. Early layers map the basic features, while the deeper layers detect higher-level features such as edges, faces, and objects. Features are represented by a number of parameters, and pooling layers are used to reduce the number of parameters without affecting the feature representation.

**Figure 2:**
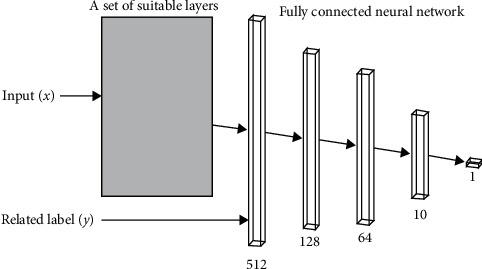
A general architecture of the noise discriminator.

Figure 2 shows a general architecture of the proposed noise discriminator. The model architecture consists of a set of convolutional and pooling layers, which is changeable according to the nature of the training dataset, followed by four hidden layers (512, 128, 64, and 10) of the fully connected neural network. The set of convolutional and pooling layers are left unspecified because they are problem-dependent.

## 4. Evaluating the Discriminator

To evaluate the proposed model's performance in identifying outliers, the MNIST, CIFAR-10 [18], and CIFAR-100 [18] datasets were used. A brief description of each dataset is provided in Table 2.

The experiments undertaken in this section were divided into four main parts: the first part examined the discriminator's behavior in identifying outliers; the second part verified the effectiveness of the proposed method in terms of the achieved classification accuracy of classifiers trained using the cleaned datasets compared to other methods reported in the literature; the third part investigates the effect of the discriminator on training time; and the last part compares different methods in terms of their accuracy in identifying outliers.

The discriminator outputs a value of 1 for genuine instances and a value of 0 for outliers, and to decrease the number of genuine instances that might be incorrectly classified as outliers, we used a threshold of 0.75, instead of 0.5. This reduces the number of false negatives; however, it may also increase the number of false positives as a side effect.

### 4.1. Evaluating the Ability of the Discriminator in Identifying Outliers

This section investigates the ability of the proposed discriminator to identify outliers in a noisy dataset. For each noisy dataset, three discriminators were trained on different datasets, $\tilde{D}$, generated using a various number of seeds (trusted instances). Each discriminator's performance was evaluated by verifying the extent to which it could distinguish between outliers and genuine instances in a noisy dataset.

The outliers were created by flipping the classes of randomly selected instances. The discriminators for MNIST, CIFAR-10, and CIFAR-100 datasets were trained for 50 epochs; after that, they were used to clean datasets with different noise ratios: 10%, 50%, and 90%.

#### 4.1.1. Evaluation on MNIST

**Figure 3:**
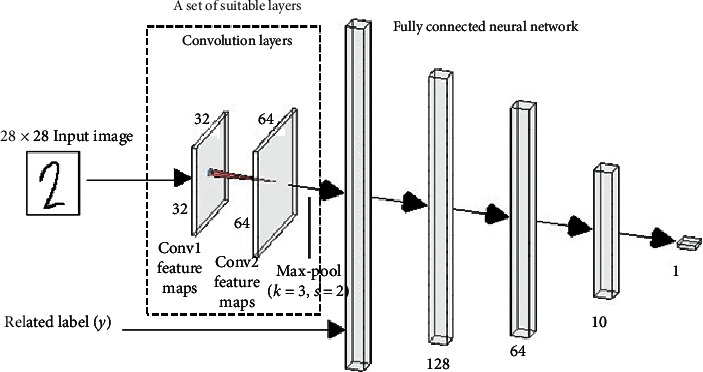
The architecture of the discriminators used for MNIST.

To implement the discriminator so as to identify the outliers added to the MNIST dataset, two convolution layers (32 and 64), in addition to a max-pooling layer, were used as shown in Figure 3.

Three discriminators were trained for MNIST, each of which was trained on a different dataset. The dataset of the first was generated using five seed instances per class with no data augmentation, whereas the datasets for the second and third were generated using two and five seed instances per class and with data augmentation. The augmentation methods we used are rotation, shifting, shearing, and zooming.

The first dataset contained 900 instances.  Table 3 summarizes the results. It reveals that the discriminator identified most outlier instances with a low false-negative rate. In addition, the false-positive rate decreases as the noise ratio increases which is expected because as the number of outliers increases, the likelihood that the discriminator is correct when it classifies an instance as an outlier also increases. This observation also justifies the inverse correlation identified between the overall recall values and the noise ratio. However, the false-negative rate was found to increase as we increase the noise ratio.

Table 4 summarizes the results we obtained using the second discriminator, which is based on two seed instances and with data augmentation. The generated training set contained 36,000 instances. The results show that this discriminator outperformed the first one, which shows the usefulness of using the augmentation operations.

For the third discriminator (i.e., based on 5 seed instances and data augmentation), $\tilde{D}$ contained 90,000 instances. This noise discriminator outperformed both the first and second discriminators, as can be seen in Table 5. With a noise ratio of 90%, this discriminator identified 97.56% of the outliers with a 1.8% false-positive rate.

A comparison of Tables 3–5 indicates that the applied data augmentation methods positively influenced discriminator performance. Although 90% is a high noise ratio, the first, second, and third discriminators identified almost all outliers with false-negative rates of 12.9%, 8.9%, and 8.77%, respectively. Furthermore, with 10% and 50% noise, the discriminators identified a reasonable number of outliers with a false-negative rate of less than 2%.

A noteworthy point for high noise ratios in MNST (i.e., 50% and 90%) is that the discriminator performance was more effective compared to low noise ratios (10%). This result stands to reason because when the amount of noise in a training dataset is substantial, the likelihood of the false positive decreases.

#### 4.1.2. Evaluation on CIFAR-10

The noise discriminator we trained for this dataset contained a 20-layer ResNet where each convolutional layer is of 16 × 16 × 3. It was shown that ResNet models are very effective for feature extraction for this dataset [28].

We trained three noise discriminators for the CIFAR-10 dataset using a different number of seed instances with data augmentation.

The first discriminator was based on 100 seed instances per class with an augmentation factor of 5. Thus, according to equation (2), the total number of instances used to train the discriminator amounted to 450,000. For the second discriminator, 200 seed instances were used per class with an augmentation factor of 2. Therefore, the total number of instances used to train the discriminator was 360,000. As for the third discriminator, 400 seed instances were used per class with an augmentation factor of 1. Hence, 360,000 instances were used to train the discriminator.

The results of the first, second, and third discriminators are given in Tables 6–8, respectively. The results clearly indicate that there is a proportional relationship between the number of seed instances used and the performance as the performance in terms of the *F*1 measure improves as we increase the number of seed instances regardless of the noise ratio.

#### 4.1.3. Evaluation on CIFAR-100

Similarly, for this dataset, we also used a 20-layer ResNet model for feature extraction, where each convolutional layer is of 16 × 16 × 3.

We also trained three discriminators for CIFAR-100 using a different number of seeds for generating the dataset to train each discriminator; data augmentation was used in all cases. The first discriminator was trained using a dataset generated based on 10 seed instances per class and with an augmentation factor of 1. Therefore, the total number of instances used to train the discriminator was 792,000. The second discriminator was trained based on 30 seed instances per class with an augmentation factor of 1. Hence, the total number of instances used to train the discriminator was 1,188,000. As for the third discriminator, 50 seed instances were used per class to generate the dataset with an augmentation factor of 1. The only augmentation method used in this case was image rotation. The total number of instances used to train the discriminator amounted to 1,980,000. Of course, the large number of instances used to train these discriminators is due to the large number of classes in CIFAR-100.

The results of the three discriminators are summarized in Tables 9–11, respectively. It is clear that the discriminator that was based on 50 seed instances outperformed its counterparts in outlier identification. With 50% noise, the 50-seed discriminator identified outlier instances with a false-negative rate of 4.87%. In general, the *F*1 measure improves as the number of seed instances and noise ratio increase.

Finally, it is worth noting that, for all the experimental evaluations undertaken in this section, the false-negative rate was reasonable, while the false-positive rate was comparatively high. This is probably because we used a relatively high threshold value of 0.75, which made the discriminators very conservative in classifying instances as genuine and less so when it comes to classifying the instances as outliers. This accounts for the high rate of false positives observed in these experiments.

### 4.2. Classification Using the Cleaned Datasets

In this section, we compare different methods for noise handling indirectly by training classifiers using the cleaned datasets obtained by each method for the original classification problem. We compare the performance of the proposed noise discriminator against the results of the methods reported in [29]. To conduct a valid performance comparison in terms of mitigating the effect of noise, the model's architecture used in [29] was adopted, along with the data corruption procedures.

**Figure 4:**
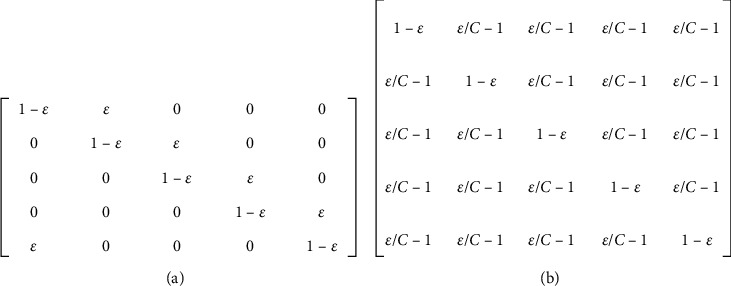
Definition of the noise transition matrix for 5 classes as an example. *ε* stands for the noise ratio, and *C* is the number of classes. (a) Transition matrix of pair flipping. (b) Transition matrix of symmetry flipping.

For dataset corruption, two types of noise transition matrices were used: pair flipping [29] and symmetry flipping [30]. Pair flipping involves changing the classes of some randomly selected instances to another speciﬁed class, according to a transition matrix (Figure 4(a)). Each column in the matrix represents a class, and the likelihood that an instance keeps its current class is 1 − *ε*, and with *ε* probability, it may change its class to the next class in the transition matrix. By contrast, symmetry flipping changes the class of a selected instance to any other class, where all classes are equally likely to be selected as a replacement of the original class, according to the transition matrix shown in Figure 4(b). As in [29], the noise ratios were 20% and 50% for symmetry noise, i.e., *ε*={0.2, 0.5}, and 45% for pair noise type, *ε*=0.45. All experiments were implemented using a 9-layer CNN trained for 200 epochs.

We compared our noise discriminator method with seven other methods for noise handling. These methods are as follows:

- Bootstrapping [9]: fixes noisy labels using the labels predicted by a neural network, where the consistency of prediction is a weighted measure for correct labels.
- S-model [31]: detects noisy instances on the basis of the constitution of a noisy transition matrix, which is used by an additional softmax layer.
- F-correction [32]: corrects a network's prediction using a noise transition matrix, where the estimation of the matrix occurs with a standard network that undergoes initial training.
- Decoupling [33]: leverages two classifiers and updates the parameters based on the samples that have predictions that contrast with the classifiers.
- MentorNet [34]: trains two networks, one a teacher and another a student. The purpose of the teacher network is for pretraining and filtering noisy instances. In turn, the output of the teacher network is used as an input for the student network for training, which is employed later for classification.
- Coteaching [29]: relies on a pair of simultaneously trained networks, each of which feeds its counterpart with supposedly clean instances during training. In this case, clean instances are defined based on their small-loss values.
- EBF [23, 24]: it is based on monitoring and analyzing the distribution of exponential moving average (EMA) values of the loss values of the training instances. An instance that continues to have a large EMA is identified as an outlier and eliminated.

All of the discriminators trained in Section 4.1 were used as a preprocess to clean the datasets. Then, a 9-layer CNN was trained using the cleaned data for the corresponding classification problem.

#### 4.2.1. Evaluation Using MNIST

Table 12 summarizes the results of all methods in terms of the classification accuracy for digit recognition using the cleaned version of the noisy MNIST. We used three noise discriminators based on two seed instances with data augmentation (2S + DA), five seed instances without data augmentation (5S), and five seed instances with data augmentation (5S + DA).

The results clearly indicate that noise negatively impacted all methods, and the accuracy decreases as the noise ratio increases. However, training a classifier using the cleaned data by the proposed discriminator enabled the model to achieve reasonable performance regardless of the noise type and noise ratio.

Under a 20% symmetry noise, every method achieved a reasonable classification accuracy. However, this accuracy decreased with a 50% noise ratio. Notably, the best-performing discriminators, which yielded overall accuracies of 96.33% and 93.07%, were the discriminator based on 5 seed instances with data augmentation and the discriminator based on 2 seed instances with data augmentation, respectively. However, they came after EBF, which achieved 98.27%.

Pair flipping represents the hardest type of noise compared to symmetry flipping, as can be seen from the results of all methods. However, all of the proposed noise discriminators outperformed all other methods. In particular, the discriminator based on five seed instances was the best, where it achieved an overall accuracy of 97.13% (approximately 9% higher than EBF, which is the second top-performing method).

It is noteworthy that the data augmentation procedure displayed a considerable improvement in terms of discriminator performance. This can be seen in Table 12, where the discriminator based on 2 seed instances for each class with data augmentation outperformed the discriminator based on 5 seed instances and without data augmentation.

#### 4.2.2. Evaluation Using CIFAR-10

The three discriminators built for CIFAR-10 in Section 4.1.2 were used to clean the noisy datasets of CIFAR-10.  Table 13 summarizes the accuracy of all methods. The table reveals that the proposed discriminators are competitive with the other methods. Moreover, the three discriminators outperformed their counterparts for pair noise.

Table 13 shows that the discriminator based on 400 seeds (400S) outperformed all other methods by a large margin with 50% symmetry noise and 45% pair noise. Specifically, the discriminator outperformed the second-best method by approximately 5% under 50% symmetry noise; and it outperformed the second-best method by approximately 8% under 45% pair noise. For 20% symmetry noise, EBF and the discriminator outperformed the other methods by achieving 85.58% and 84.72%, respectively.

#### 4.2.3. Evaluation Using CIFAR-100

The number of classes that a dataset contains is a major factor that influences the performance of all noise mitigation methods. The higher this number is, the more challenging the dataset is. Therefore, the CIFAR-100 dataset, which contains 100 classes, represented a significant challenge for all methods.

In this experiment, the three discriminators built for CIFAR-100 in Section 4.1.3 which were based on 10, 30, and 50 seeds per class were used to clean the noisy CIFAR-100 dataset.  Table 14 summarizes the results of all methods.

As shown in Table 14, for 45% pair noise, the discriminator based on 50 seeds (50S) outperformed all other methods by at least 12% higher accuracy. It also came in second place (after EBF) for 50% symmetry noise. However, EBF accuracy was drastically decreased for 45% pair noise.

### 4.3. The Effect of the Discriminators on Training Time

This section investigates the impact of the discriminator on the training time of the classifiers that were trained using the cleaned datasets. We will also study its impact on the number of epochs and the achieved classification accuracy under specific conditions. Several experiments were undertaken on noisy versions of the MNIST, CIFAR-10, CIFAR-100, Fashion [35], and Traffic Signs [36] datasets, which were used to train a 9-layer CNN before and after cleaning the datasets with the proposed discriminator. It is intuitive that if the discriminator effectively eliminates outliers, the training time and the number of epochs needed to train the classifiers will decrease while increasing the classification accuracy.

Rather than training the classifiers for a specific number of training epochs, as we did so far, in the following experiments, the training process will continue until one of the following two conditions is met: either the training loss (error) value falls below 0.03 or when a maximum of 300 epochs is reached. In each experiment, we recorded the training time.

Given that three discriminators were trained for each dataset in Section 4.1, we used the discriminator that gave the best classification accuracy for every dataset, namely, for MNIST, we used the discriminator based on five seeds, while for CIFAR-10 and CIFAR-100, we used the discriminators based on 400 and 50 seeds, respectively.

In this section, we also use two more datasets: Fashion and Traffic Signs. Fashion dataset includes 60,000 instances in the training set and 10,000 instances in the test set. Each instance is a 28 × 28 gray image and labeled with one of ten classes. The Traffic Signs dataset contains 34,799 instances for training purposes and 12,630 instances for testing purposes. Each instance is represented as an RGB image of 32 × 32 and is labeled by one of 43 classes. For the Fashion dataset, we trained a discriminator based on ten seeds with data augmentation. We used an augmentation factor of ten, which means that the discriminator was trained using 90,000 instances (see equation (2)). The architecture of the discriminator model for Fashion is the same as the model used for MNIST. Finally, for the Traffic Signs dataset, we trained a discriminator using ten seeds with data augmentation. Due to the large number of classes in this dataset (43 classes), we used a small augmentation factor of one, which implies that the discriminator was trained on 180,600 instances (see equation (2)). The discriminator model's architecture was the same as the model used for CIFAR-10.

Table 15 shows that, after using the proposed discriminator to clean the noisy datasets and using the cleaned data to train classifiers, a substantial improvement was observed in classification accuracy and training time compared to training the classifiers using the noisy (uncleaned) dataset. The only exception was in the Fashion dataset with 20% symmetric noise. The use of the discriminator led to a reduction in accuracy by 1.42%, but the training time was also reduced in this experiment too.

A remarkable improvement in classification accuracy was observed for the MNIST dataset after using the discriminator to clean the data, along with improvements in the required number of training epochs and the required training time. Notably, a considerable improvement was achieved for the MNIST results as a result of using the discriminator, as can be seen in Table 15.

As for CIFAR-10, Table 15 shows that a considerable improvement in classification accuracy was achieved for all types of noise. However, with 20% symmetric noise, a slight increase of two training epochs was noted, but still, the training time was reduced from 8.2 K seconds down to 5.1 K. The reduction in training time is due to the reduction in the size of the training data (as a result of eliminating the noisy instances). Also, in the case of 45% pair noise, the number of training epochs grew from 89 to 129, but still, the training time was reduced from 8.6 K down to 5.3 K seconds.

Regarding the CIFAR-100 dataset, the maximum number of training epochs (300) was reached in all experiments. However, a substantial improvement occurred in classification accuracy and training time in all experiments. The reduction in training time is due to the reduction in the size of the training data, which was reduced from 50,000 instances to 16,701, 10,920, and 11,917 instances in case of 20% symmetric noise, 50% symmetric noise, and 45% pair noise, respectively.

As for the Fashion dataset, training the classifiers using the cleaned data considerably improved the classification accuracy and reduced the number of training epochs and training time. The only exception is with 20% symmetry noise, where the classification accuracy was reduced from 85.76% down to 84.34%.

Regarding the Traffic Signs dataset, which contains 43 classes, using the discriminator to clean the training data resulted in considerable improvements in training time, training epochs, and classification accuracy.

The results indicate that the proposed noise discriminator method led to a substantial reduction in training time across every dataset. This comes as a result of reducing the number of training epochs, reducing training data size, or both. Furthermore, the use of the discriminator increased the classification accuracy, particularly when we have a high ratio of outliers. Hence, it is reasonable to conclude that the use of a noise discriminator to clean the training data has a positive effect on the training time, training epochs, and classification accuracy in most cases.

**Figure 5:**
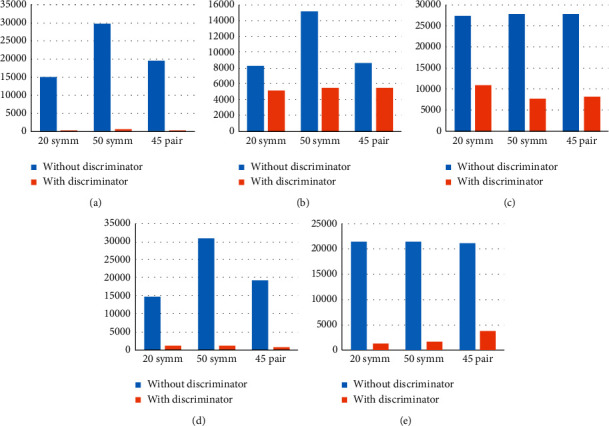
Comparison of training time with and without using the proposed discriminator: (a) MNIST, (b) CIFAR-10, (c) CIFAR-100, (d) Fashion, and (e) Traffic Signs.

It is worth noting that, in this evaluation, the training time of the discriminator was not taken into consideration. Nevertheless, Figure 5 indicates that the reduction in training time was remarkable, which compensates for the procedures undertaken as a preprocess before training. In any case, the improvement in the classification accuracy justifies the training time of the discriminator.

### 4.4. Comparing the Classification Accuracy of Different Methods

Noise type and noise ratio are critical factors that pose major challenges for the performance of noise mitigation methods. Pair flipping represents the hardest case of noise. For instance, F-correction performed reasonably under symmetry noise, while it failed completely to learn under pair noise in all datasets (see Tables 12–14). The accuracy of every method was lower for pair noise compared to symmetry noise, but by contrast, the proposed discriminator performed well under pair noise as was discussed in Section 4.2.

**Figure 6:**
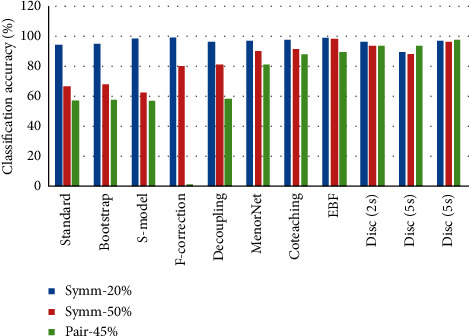
Test accuracy of each method among different types and ratios of noise on MNIST.

**Figure 7:**
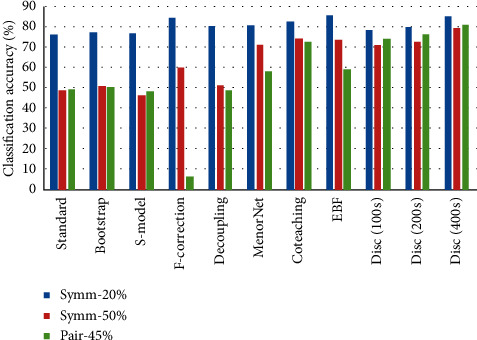
Test accuracy of each method among different types and ratios of noise on CIFAR-10.

**Figure 8:**
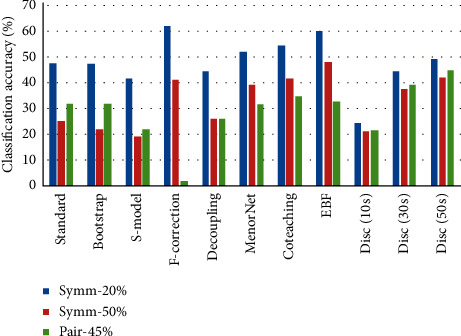
Test accuracy of each method among different types and ratios of noise on CIFAR-100.

Figures 6–8 present each method's classification accuracy results for different types and ratios of noise for all datasets. It is clear from these figures that the type and ratio of noise represent a challenge for all methods. However, the proposed noise discriminator's performance is stable irrespective of the type and ratio of noise. Therefore, it is reasonable to conclude that the proposed discriminator offers a vital improvement in terms of accelerating the training process after cleansing the training data.

Although the proposed discriminator demonstrates superior results compared to the other methods, its limitations cannot be overlooked for datasets with many classes. Training the proposed discriminator to clean a dataset with a substantial number of classes requires a large training dataset. This is because the size of the dataset generated to train a discriminator is proportional to the number of seeds and the number of classes. Therefore, if the number of classes is large, then the size of the dataset needed to train the discriminator will also be large. For a dataset of 100 classes, for instance, the discriminator may need to be trained on a dataset of 1,980,000 instances, which requires relatively processing resources. Investigating other methods to reduce this number may be an interesting future work.

## 5. Conclusion

Many machine learning methods, including deep learning models, require vast amounts of high-quality training data to perform well. However, large datasets usually contain noise, which may undermine the performance. This paper proposes a novel discriminator for outlier detection, and it presents a systematic approach for generating the dataset required for training the noise discriminators based on a small number of genuine instances. The empirical results show that the proposed method is effective, especially for training data that contain excessive noise. The results also indicate that the proposed method performs well regardless of the type of noise (e.g., pair or symmetry noise). The method was compared with seven other methods with respect to the achieved accuracy after noise mitigation.

In the future, we intend to investigate utilizing other algorithms of learning such as semisupervised learning or weakly supervised learning to take advantage of those instances which were identified as outliers by the proposed discriminator. Furthermore, we intend to investigate the use of similar discriminators to relabel the identified outliers.

## Figures and Tables

**Algorithm 1:**
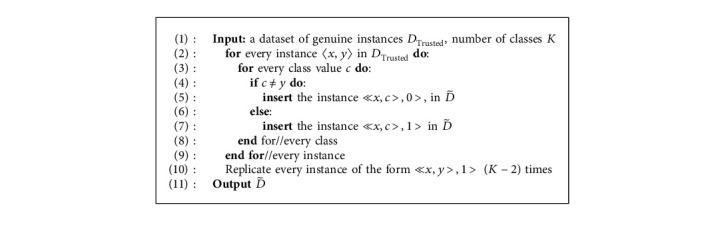
Creating dataset $\left(\tilde{D}\right)$ for training the proposed discriminator.

**Table 1:** A sample of the generated dataset for digit 2.

Input		Output(0 or 1)
*x*	*y*	
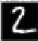	0	0
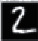	1	0
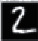	2	1 (correct)
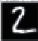	3	0
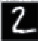	4	0
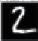	5	0
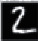	6	0
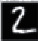	7	0
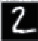	8	0
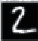	9	0

**Table 2:** Brief description of datasets.

Dataset	Dim.	Training set (K)	Testing set (K)	# classes
MNIST	28 × 28	60	10	10
CIFAR-10	32 × 32	50	10	10
CIFAR-100	32 × 32	50	10	100

**Table 3:** The results of the 5-seed discriminator for MNIST.

Noise ratio	10 (%)	50 (%)	90 (%)
Recall	68.42	80.25	95.6
*F*1 measure	38.44	83.48	97.54
False negative	0.19	1.04	12.9
False positive	76.1	28.1	3.7

**Table 4:** The results of the 2-seed discriminator for MNIST with data augmentation.

Noise ratio	10 (%)	50 (%)	90 (%)
Recall	75.68	86.17	96.64
*F*1 measure	44.93	87.68	98.06
False negative	0.12	1.1	8.9
False positive	70.9	21.3	2.88

**Table 5:** The results of the 5-seed discriminator for MNIST with data augmentation.

Noise ratio	10 (%)	50 (%)	90 (%)
Recall	84.82	93.78	97.56
*F*1 measure	56.54	93.83	98.58
False negative	0.17	1.8	8.77
False positive	60.4	9.9	1.8

**Table 6:** The results of the 100-seed discriminator for CIFAR-10.

Noise ratio	10 (%)	50 (%)	90 (%)
Recall	60.21	74.61	89.14
*F*1 measure	31.55	78.48	93.89
False negative	1.60	11.58	53.48
False positive	80.95	31.90	4.84

**Table 7:** The results of the 200-seed discriminator for CIFAR-10.

Noise ratio	10 (%)	50 (%)	90 (%)
Recall	61.42	75.97	90.23
*F*1 measure	32.60	79.61	94.53
False negative	1.27	9.63	48.97
False positive	80.25	30.86	4.77

**Table 8:** The results of the 400-seed discriminator for CIFAR-10.

Noise ratio	10 (%)	50 (%)	90 (%)
Recall	70.68	80.94	91.03
*F*1 measure	38.98	83.09	94.94
False negative	1.02	8.46	45.91
False positive	75.39	25.35	3.63

**Table 9:** The results of the 10-seed CIFAR-100 discriminator.

Noise ratio	10 (%)	50 (%)	90 (%)
Recall	20.05	55.36	90.63
*F*1 measure	19.91	69.02	95.03
False negative	0.63	4.70	31.46
False positive	88.94	47.15	8.98

**Table 10:** The results of the 30-seed CIFAR-100 discriminator.

Noise ratio	10 (%)	50 (%)	90 (%)
Recall	44.29	67.90	91.49
*F*1 measure	25.91	75.20	95.37
False negative	0.74	6.49	38.06
False positive	85.06	38.73	6.55

**Table 11:** The results of the 50-seed CIFAR-100 discriminator.

Noise ratio	10 (%)	50 (%)	90 (%)
Recall	46.95	69.58	92.21
*F*1 measure	27.04	76.29	95.76
False negative	0.46	4.87	31.80
False positive	84.33	37.50	6.24

**Table 12:** Average test accuracy on MNIST over the last ten epochs. The top part of the table presents the results that were adapted as published in [29]. The bottom part shows the results achieved by our implementation.

Method	Flipping rate		
	Symmetry-20%	Symmetry-50%	Pair-45%
Standard	94.05 (±0.16)	66.05 (±0.61)	56.52 (±0.55)
Bootstrap	94.40 (±0.26)	67.55 (±0.53)	57.23 (±0.73)
S-model	98.31 (±0.11)	62.29 (±0.46)	56.88 (±0.32)
F-correction	98.80 (±0.12)	79.61 (±1.96)	0.24 (±0.03)
Decoupling	95.70 (±0.02)	81.15 (±0.03)	58.03 (±0.07)
MentorNet	96.70 (±0.22)	90.05 (±0.30)	80.88 (±4.45)
Coteaching	97.25 (±0.03)	91.32 (±0.06)	87.63 (±0.21)

EBF	**98.75 (±0.29)**	**98.27 (±0.39)**	88.91 (±0.62)
Discriminator (2S + DA)	96.27 (±0.20)	93.07 (±0.73)	93.29 (±0.32)
Discriminator (5S)	89.12 (±0.23)	87.67 (±0.51)	93.26 (±0.20)
Discriminator (5S + DA)	96.98 (±0.34)	96.33 (±0.36)	**97.13 (±0.18)**

**Table 13:** Average test accuracy on CIFAR-10 over the last ten epochs.

Method	Flipping rate		
	Symmetry-20%	Symmetry-50%	Pair-45%
Standard	76.25 (±0.28)	48.87 (±0.52)	49.50 (±0.42)
Bootstrap	77.01 (±0.29)	50.66 (±0.56)	50.05 (±0.30)
S-model	76.84 (±0.66)	46.15 (±0.76)	48.21 (±0.55)
F-correction	84.55 (±0.16)	59.83 (±0.17)	6.61 (±1.12)
Decoupling	80.44 (±0.05)	51.49 (±0.08)	48.80 (±0.04)
MentorNet	80.76 (±0.36)	71.10 (±0.48)	58.14 (±0.38)
Coteaching	82.32 (±0.07)	74.02 (±0.04)	72.62 (±0.15)

EBF	**85.58** (±0.58)	74.30 (±1.26)	59.17 (±1.91)
Discriminator (100S)	78.09 (±0.90)	70.84 (±0.81)	74.24 (±1.31)
Discriminator (200S)	79.79 (±0.61)	72.44 (±1.93)	76.33 (±1.15)
Discriminator (400S)	84.72 (±0.53)	**79.05 (±0.72)**	**80.57 (±1.21)**

**Table 14:** Average test accuracy on CIFAR-100 over the last ten epochs.

Method	Flipping rate		
	Symmetry-20%	Symmetry-50%	Pair-45%
Standard	47.55 (±0.47)	25.21 (±0.64)	31.99 (±0.64)
Bootstrap	47.00 (±0.54)	21.98 (±6.36)	32.07 (±0.30)
S-model	41.51 (±0.60)	18.93 (±0.39)	21.79 (±0.86)
F-correction	**61.87 (±0.21**)	41.04 (±0.07)	1.60 (±0.04)
Decoupling	44.52 (±0.04)	25.80 (±0.04)	26.05 (±0.03)
MentorNet	52.13 (±0.40)	39.00 (±1.00)	31.60 (±0.51)
Coteaching	54.23 (±0.08)	41.37 (±0.08)	34.81 (±0.07)

EBF	59.90 (±0.66)	**48.51** (±0.61)	32.65 (±0.60)
Discriminator (10S)	24.45 (±0.83)	20.93 (±0.62)	21.42 (±0.88)
Discriminator (30S)	44.36 (±0.47)	37.70 (±0.67)	39.26 (±0.81)
Discriminator (50S)	49.31 (±0.67)	41.79 (±1.11)	**44.62 (±0.54)**

**Table 15:** Effect of the discriminator model on the training time.

		20% symm. noise			50% symm. noise			45% pair noise		
		Test acc.	Time (sec)	Epochs	Test acc.	Time (sec)	Epochs	Test acc.	Time (sec)	Epochs
MNIST	Standard	93.15	15.1 K	148	64.41	29.8 K	300	54.39	19.5 K	197
	Discriminator	95.60	273.43	4	94.83	523.97	12	96.02	198.38	4

CIFAR-10	Standard	71.38	8.2 K	90	47.50	15.2 K	158	47.58	8.6 K	89
	Discriminator	83.06	5.1 K	92	78.69	5.4 K	139	80.00	5.3 K	129

CIFAR-100	Standard	37.78	27.3 K	300	16.60	27.7 K	300	27.63	27.7 K	300
	Discriminator	50.78	10.8 K	300	42.46	7.6 K	300	44.69	8.2 K	300

Fashion	Standard	85.76	14.7 K	145	56.93	30.3 K	300	54.67	19.3 K	190
	Discriminator	84.34	1.1 K	19	79.58	1.1 K	29	85.09	793.46	18

Traffic Signs	Standard	88.55	21.3 K	300	73.64	21.4 K	300	51.44	21.2 K	300
	Discriminator	92.95	1.3 K	21	92.12	1.7 K	51	91.96	3.7 K	101

K refers to a thousand of seconds.

## Data Availability

The data that were used in the manuscript are the benchmark data that are already cited.
